# Language, Social Support, and Recovery-Stage Transitions in Opioid Use Disorder on Reddit: Computational Analysis

**DOI:** 10.2196/91054

**Published:** 2026-09-17

**Authors:** Xinchen Yu, Huai-yu Chen, Yu Chi

**Affiliations:** 1 Department of Computer Science, University of Arizona, Tucson, AZ, United States; 2 Department of Communication, National Chengchi University, Taipei, Taiwan; 3 Department of Health Education and Health Promotion, College of Education, National Taiwan Normal University, Taipei, Taiwan; 4 School of Information, College of Information, Data and Society, San Jose State University, 1 Washington Sq, San Jose, CA, 95192, United States, 1 4125396621

**Keywords:** opioid use, recovery stages, social support, deep learning, Reddit, social media

## Abstract

**Background:**

Recovery from opioid use disorder (OUD) is a complex, nonlinear process involving substantial health, psychological, and social challenges. Although online social support has been shown to benefit individuals with OUD, less is known about how recovery stages, such as the initial or stable stages, are expressed and experienced in online communities. Specifically, the linguistic features characterizing each stage, the social support exchanged at each stage, and the feasibility of predicting these stage transitions from user-generated content remain largely unexplored.

**Objective:**

This study aimed to develop a computational framework and present empirical insights for understanding OUD recovery in online communities by characterizing the language individuals use at different stages, the social support they receive, and the transitions they undergo over time.

**Methods:**

We collected 32,810 posts and 324,224 comments from *r/OpiatesRecovery*, the largest Reddit community dedicated to opioid recovery from 2014 to 2022. We fine-tuned pretrained language models to classify posts into 5 recovery stages and identify 11 categories of social support in comments. Recovery trajectories were constructed for 2936 users who posted multiple times. Mann-Whitney *U* tests and one-way multivariate analysis of covariance were used to compare linguistic features and social support across recovery stages and transitions. In addition, we predicted recovery-stage transitions using fine-tuned RoBERTa (Robustly Optimized BERT Pretraining Approach) and open-source large language models (Llama-3.1-8B-Instruct and Qwen2.5-14B-Instruct) evaluated in zero-shot and few-shot settings.

**Results:**

Individuals in early-stage recovery used significantly more negative, painful, and passive language compared to those in later stages (*P*<.001). They also received more informational support (eg, advice and factual guidance) but less emotional support (eg, encouragement and sympathy; *P*<.001). Notably, posts followed by observed recovery-stage progression were associated with significantly more informational support than posts followed by no observed stage change (*P*<.001). Regarding the prediction of individuals’ future recovery transitions, fine-tuned RoBERTa outperformed prompted open-source large language model baselines in this benchmark (*F*_1_-score=0.59 vs 0.43), although this task remained highly challenging.

**Conclusions:**

This study reveals distinct linguistic and social support patterns across OUD recovery stages, identifying an association between informational support and observed recovery-stage progression. Although automatically detecting stage transitions remains challenging, these findings may inform future research on timely, stage-appropriate support strategies in online recovery communities.

## Introduction

### Background

Opioid use disorder (OUD) is a chronic condition requiring long-term management. It changes people’s brains, behavior, and motivational hierarchy; reduces their career opportunities; increases the risks of other diseases; and often ends in death [1]. Opioid-involved overdose deaths remain at high levels in the United States. In 2023, an estimated 5.7 million people aged ≥12 years had OUD, according to the National Survey on Drug Use and Health [2]. A recent report from the Centers for Disease Control and Prevention estimated that 87,000 drug overdose deaths occurred in the United States between October 2023 and September 2024 [3]. One well-accepted strategy for addiction management is joining and participating in social support groups wherein individuals with shared or similar experiences offer mutual support [4]. In contrast to professional clinical guidance, which is typically expert-driven, social support is rooted in communal background and shared experiences. This foundation can foster a deeper sense of trust, understanding, and acceptance among individuals [5].

Moreover, the rise of social media and microblogging platforms has facilitated the emergence of online health communities that offer accessible spaces for advice and support. The anonymity afforded by these online platforms, such as Reddit, enables users to engage in discussions on sensitive topics with reduced concern about stigma or judgment. Prior work shows that people affected by stigmatized conditions are more likely to seek help from peers and seek help online [6,7]. In the mental health domain, for example, online social support has already been shown to be effective in helping individuals with substance use conditions [8] as well as motivating peers to engage more consistently with treatment [9].

Social support is generally considered to be a multidimensional construct with several distinct classes or types [10]. A meta-analysis showed that informational and emotional support are the 2 most commonly observed types in online health communities [11]. Informational support refers to advice and factual feedback intended to help a recipient better understand and respond effectively to their problems [10], and emotional support refers to messages or actions assuring individuals that they are cared for, loved, esteemed, and valued [12]. By virtue of the shared experiences, online community members can be ideal providers of informational and emotional support. For example, in cancer online support groups, research has suggested that informational support improves psychological well-being [13] and emotional support helps patients adjust to the stress of living with and fighting against their diseases and serves as an outlet for users’ emotional needs [14,15].

Existing literature on leveraging user-generated web content to study OUD has examined the behaviors and characteristics of support seekers, such as their recovery stages [16], self-disclosure [17,18], and information-seeking behaviors [19]. Other studies have focused on support providers, analyzing the types and frequency of social support [20]. However, large-scale studies associating recovery stages of support seekers with the types of social support they receive remain limited. While small-scale user studies have demonstrated that online social support is especially beneficial during the early stages of recovery [21], few have examined how recovery stages evolve over time for the same individuals in online health communities. We argue that recovery, particularly in the context of OUD, is a dynamic rather than a static process: a person may claim to be in stable recovery at one point but later report a relapse. Tracing transitions between recovery stages allows us to capture the early signals of future recovery trajectories and examine how different types of social support are associated with observed recovery stage changes over time.

### Objectives

Motivated by the limitations of existing studies, this work aimed to investigate how individuals’ recovery stages from OUD, and their transitions between stages over time, are related to the types of social support they receive in web-based recovery communities. To this end, we developed and applied a theoretically grounded computational framework that integrates linguistic, social support, and longitudinal trajectory perspectives, applied to posts and comments from *r/OpiatesRecovery*, the largest Reddit community dedicated to opioid recovery. Specifically, we ask the following research questions (RQs):

- RQ1. What linguistic features characterize and distinguish posts across different stages of recovery?
- RQ2. How do the types of social support received in response to posts vary across different stages of recovery?
- RQ3. How is the social support received on a post associated with the author’s subsequent recovery-stage transitions (eg, from addiction to initial recovery)?
- RQ4. How accurately can models predict recovery-stage transitions, and does incorporating post titles alongside post content improve performance?

## Methods

### Research Framework

**Figure 1.**
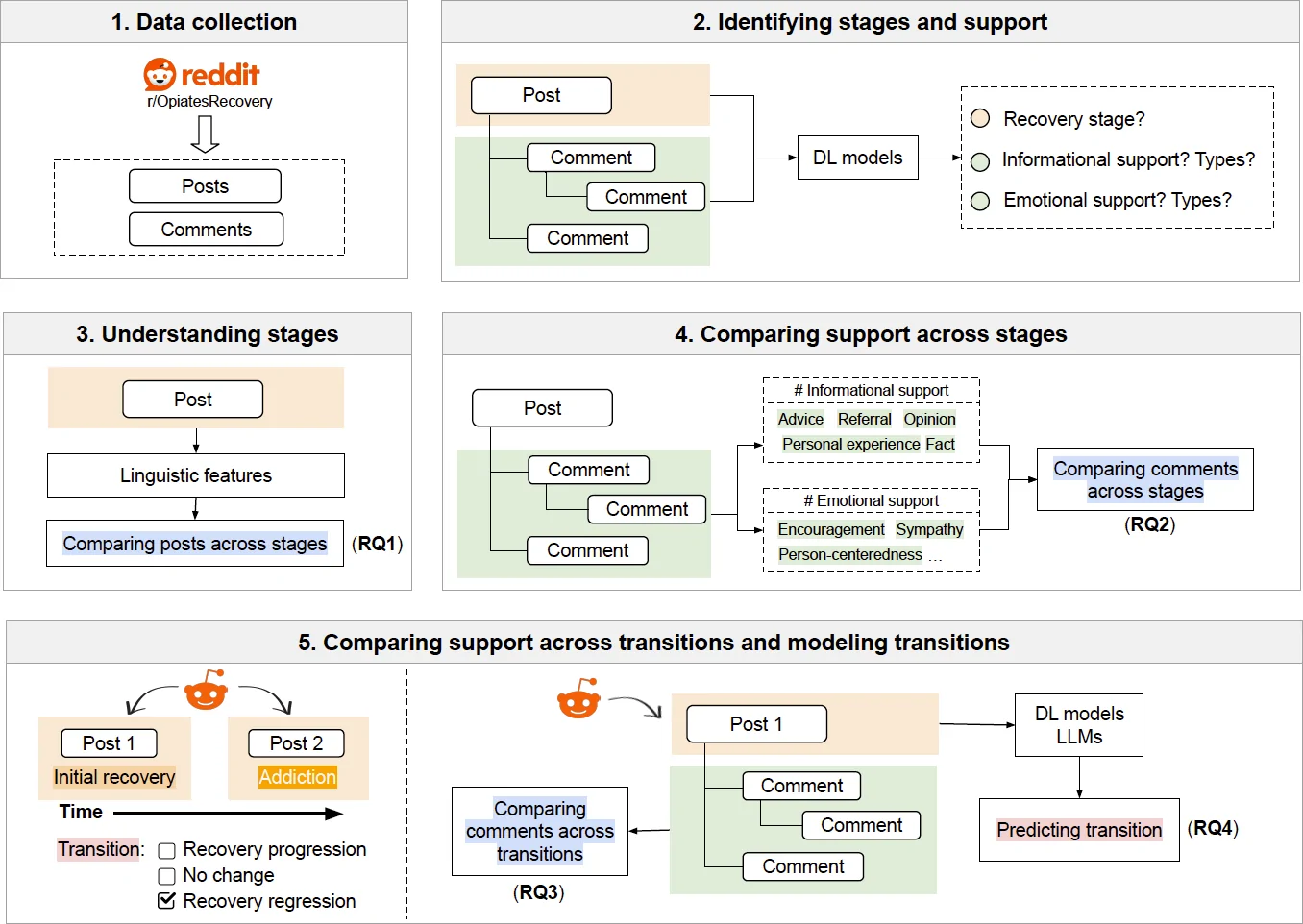
Research framework for the computational analysis of recovery stages, social support, and recovery-stage transitions in opioid use disorder using Reddit posts and comments from r/OpiatesRecovery from 2014 to 2022. DL: deep learning; RQ: research question.

This study focused on the stages of recovery in OUD. Building on our prior work, which analyzed self-disclosure and informational support at the post level using a large Reddit dataset [18], this study advanced the analysis by developing a theoretically grounded computational framework (Figure 1). Guided by the framework, we first investigated the linguistic features of posts across distinct recovery stages (RQ1) and how the 11 subtypes of informational support and emotional support received varied across stages (RQ2). We further used the framework to study the user-level recovery trajectories of individuals over time as they participate in the online health community and analyzed the interplay between social support and their recovery transitions (RQ3). Finally, we conducted experiments to forecast individuals’ future transitions given the current posts they made (RQ4).

### Dataset

We chose Reddit as the starting point of our corpus and worked with data from *r/OpiatesRecovery*, the largest subreddit on Reddit that served as a supportive community for people recovering from opiate or opioid addiction. This subreddit focused on harm reduction, sobriety, and mutual encouragement among individuals who were currently in recovery, thinking about starting recovery, or supporting their loved ones through recovery.

We retrieved 32,810 posts and 324,224 comments from *r/OpiatesRecovery* using the Python Reddit API Wrapper [22]. The posts spanned an 8-year period from January 1, 2014, to May 5, 2022. Each post contained metadata, including the post ID, author username (pseudonymized), time stamp, title, and body text. Each comment included the same metadata and was linked to its corresponding parent post.

### Characterizing Posts and Comments

#### Overview

To perform large-scale analysis, we developed a structured annotation schema for labeling recovery stages in posts and social support types in comments. The schema was grounded in prior literature and informed by an initial round of manual coding. We then fine-tuned transformer-based classifiers using the annotated data and incorporated supplemental external corpora in training the models when available. Specifically, we experimented with BERT (Bidirectional Encoder Representations from Transformers)–based [23] and RoBERTa (Robustly Optimized BERT Pretraining Approach).

#### Recovery Stages in Posts

OUD recovery is often conceptualized as progressing through stages or phases, which reflects a person’s progress in their recovery journey from OUD [24,25]. In our annotation scheme, each post was labeled with 1 of 5 recovery stages: (1) *addiction* (ie, stage 1: actively using opiates), (2) *initial recovery* (stage 2: abstinence lasting for less than 1 mo), (3) *sustained recovery* (stage 3: abstinence lasting for 1 mo to 5 y), (4) *stable recovery* (stage 4: abstinence lasting for more than 5 y), and (5) *unknown stage* (cannot tell the stage or the stage is not disclosed). Two trained human annotators (YC and HC) independently labeled a random sample of 200 posts. Among these, 21 posts were excluded because the original content had been deleted by users. The remaining posts achieved a Cohen κ of 0.65 and a percent agreement of 74.3%, reflecting moderate reliability [26]. Among the 46 initial disagreements, 21 (45.7%) involved adjacent recovery stages, 19 (41.3%) involved a specific stage versus unknown, and 6 (13.1%) involved nonadjacent stages, suggesting that disagreement was concentrated in boundary cases and ambiguous stage disclosure; details are provided in Multimedia Appendix 1.

The fine-tuned BERT-based classifier achieved *F*_1_-scores ranging from 0.69 to 0.89 across the recovery stage categories. Among the 4 recovery stages included in downstream analyses, performance was lowest for initial recovery (*F*_1_-score=0.69) and higher for addiction, sustained recovery, and stable recovery (*F*_1_-score=0.89 for each). The unknown stage category, which was excluded from downstream stage and transition analyses, achieved an *F*_1_-score of 0.73. Detailed model configurations and per-class precision, recall, and *F*_1_-scores are provided in Multimedia Appendix 1.

#### Presence and Types of Social Support in Comments

To further characterize the social support within the community, we examined each comment to determine whether it provided informational support or emotional support, the 2 most commonly exchanged forms of support in online health communities [10,27], and what specific type of support was provided.

Each comment providing informational support was further labeled with one or more of the following subtypes [28]: (1) *advice*, whether the comment offered suggestions or strategies for coping with challenges; (2) *referral*, whether the comment directed the user to external resources such as books, websites, or support groups; (3) *fact and situational appraisal*, whether the comment presented factual information or offered a reevaluation of the poster’s situation; (4) *personal experience*, whether the comment shared personal stories or incidents; and (5) *opinion*, whether the comment expressed a personal viewpoint or belief not necessarily grounded in fact.

We chose pretrained BERT-based models fine-tuned on task-specific annotated datasets to classify informational support subtypes. The models demonstrated *F*_1_-scores ranging from 0.78 to 0.94 across informational support categories, with the lowest performance for *fact and situational appraisal* (*F*_1_-score=0.78). Detailed model configurations and per-label precision, recall, and *F*_1_-scores are provided in Multimedia Appendix 1.

For emotional support, we identified 6 subtypes, which were selected based on both theoretical foundations and the availability of labeled corpora for training. Each comment was classified into one or more of the following subcategories: (1) *encouragement*, whether the comment aimed to uplift or motivate the Reddit user who posted [29]; (2) *sympathy*, whether it expressed concern, sorrow, or compassion [29]; (3) *person-centeredness*, whether it acknowledged and validated the poster’s unique perspective [11]; (4) *emotional reaction*, whether it reflected affective responses to the poster’s experience [30]; (5) *interpretation*, whether it attempted to explain or reframe the poster’s emotions [30]; and (6) *exploration*, whether it invited the poster to reflect further on their thoughts or feelings [30]. We chose pretrained RoBERTa-based models [31] fine-tuned on task-specific datasets to classify each emotional support subtype. *F*_1_-scores for the emotional support classifiers ranged from 0.60 to 0.96 across categories, with lower performance for sympathy (*F*_1_-score=0.60) and encouragement (*F*_1_-score=0.62). Detailed model configurations and per-label precision, recall, and *F*_1_-scores are provided in Multimedia Appendix 1.

### Linguistic Analyses

We studied the linguistic characteristics of posts across various recovery stages, shedding light on the differences between the language used by the Reddit users in different recovery stages. We analyzed sentiment and cognitive factors using the Sentiment Analysis and Social Cognition Engine (SEANCE) lexicon, a widely recognized tool for psychological linguistic analysis [32]. We ran the Mann-Whitney *U* test [33]. We also reported whether each feature passed the Bonferroni correction, as multiple hypothesis tests were conducted.

### Social Support Across Different Recovery Stages

We first conducted a Pearson correlation to examine associations among different types of social support. Next, we conducted one-way ANOVA to test the differences in each type of informational and emotional support across the 4 recovery stages (ie, *addiction, initial recovery, sustained recovery, and stable recovery*). Games-Howell tests were conducted for the post hoc comparisons across groups to address the violation of the assumption of homogeneity. To control variation in comment counts across posts, we normalized each social support subtype by the total number of comments per post. This approach converted raw counts into proportions, ensuring that posts with various numbers of comments remained comparable and did not disproportionately influence the analysis.

### Recovery-Stage Transitions and Social Support

To examine how the social support individuals received during their current stages of recovery was associated with subsequent recovery-stage transitions, we collected all posts in the *r/OpiatesRecovery* from 2014 to 2022. To track Reddit users’ transitions from one recovery stage to another, we excluded posts where the recovery stages could not be identified (ie, *unknown stage*). In total, 2936 unique authors had made at least two posts. We paired each post with its immediate successor in chronological order, designating them as the start and end posts. This process yielded a total of 8070 post pairs. The temporal spacing between consecutive posts varied across users, ranging from 0 to 2731 (mean 69.64, SD 169.69) days. The distribution was positively skewed, with 49.4% of post pairs occurring within 10 days. As longer intervals could allow greater opportunity for recovery stage change and greater comment accumulation, the posting interval was included as a covariate in subsequent analyses to account for temporal heterogeneity across post pairs. We further assigned a transition label to each post pair based on changes in the recovery stages. If the stage in the end post reflected progress from the stage in the start post (eg, from addiction to sustained recovery), the label was *Recovery Progression*. If it indicated a setback (eg, a return to addiction), the label was *Recovery Regression*. If there was no change in the stages between the 2 posts, the label was *No Change*. The distribution of transition labels was as follows: 1936 (24%) *Recovery Progression*, 4448 (55.1%) *No Change*, and 1686 (20.9%) *Recovery Regression*.

Using the labels obtained by our classifiers, we conducted a one-way multivariate analysis of covMultivariate Analysis of Covariance (MANCOVA) to examine differences in the types of social support in the comments as well as the linguistic features of posts across *Recovery Progression*, *No Change*, and *Recovery Regression*. To control the impact of varying time intervals on comment volume, we included time interval as a covariate in our MANCOVA models.

The test of equality of covariance matrices (Box M=988.75; *P*<.001) suggested significant differences in the covariance structure among groups. Results from the Levene tests indicated significant variance differences for *word counts* (*F*_2,7484_=31.09; *P*<.001), *fact* (*F*_2,7484_=8.34; *P*<.001), *encouragement* (*F*_2,7484_=3.56; *P*=.03), and *positive tones* (*F*_2,7484_=4.13; *P*=.02), suggesting a violation of the assumption of equal error variances. However, MANCOVA is generally robust to moderate departures from homogeneity when sample sizes are large [34]. Given the large sample (N=8070), these violations were unlikely to materially affect the results. In addition, Games-Howell post hoc tests were used because they do not assume equal variances. The results should therefore be interpreted with appropriate caution.

### Forecasting Recovery-Stage Transitions

#### Overview

To capture early signals of individuals’ future recovery stages based on their current posts, we experimented with models to forecast transitions (ie, *Recovery Progression*, *No Change*, and *Recovery Regression*). As the first study to automatically predict recovery transitions, our goal was to establish an initial benchmark and to examine how pretrained language models and large language models (LLMs) performed on this task. Specifically, we experimented with the off-the-shelf RoBERTa transformer [31] and 2 open-source instruction-tuned LLMs (ie, Llama-3.1-8B-Instruct [35] and Qwen2.5-14B-Instruct [36]). We first conducted a manual validation to ensure the accuracy of the ground truth labels in the test set. Next, we described our experimental setup and presented the evaluation results.

#### Manual Validation

To ensure the ground truth in the test set, we conducted an additional round of human annotations. First, we randomly selected 200 pairs of (start and end) posts, with each pair consisting of 2 posts by the same author that were consecutive in time. HC and YC manually annotated the recovery stages of both the start and end posts. We then compared the recovery stage of the start post and that of the end post to assign 1 of 3 transition labels to each pair: *Recovery Progression*, *No Change*, or *Recovery Regression*.

#### Experiments

##### Overview

We randomly split the 8070 instances, using 85% for training and 15% for validation, with stratification by transition label to preserve the class distribution across splits. As the transition labels were imbalanced, we also experimented with weighted cross-entropy loss using class weights inversely proportional to class frequency. This class-balanced training strategy yielded a weighted *F*_1_-score of 0.56 (SD 0.02) and did not outperform the best unweighted RoBERTa configuration, so we reported the unweighted model as the main result.

To investigate whether incorporating the title was beneficial, we considered three textual inputs: (1) the title of the post only (title), (2) the post body only (post), and (3) the title and post combined (title+post). We experimented with both supervised fine-tuning and LLM prompting approaches. All models were evaluated on the same held-out test set.

##### RoBERTa Fine-Tuning

We fine-tuned RoBERTa-base [31], provided by Hugging Face [37], for sequence classification with 3 output labels. Training used the AdamW optimizer with a learning rate of 1×10⁻⁵, weight decay of 0.01, and a batch size of 8, over a maximum of 5 epochs. We applied early stopping with a patience of 3 epochs and selected the best checkpoint based on validation loss evaluated at the end of each epoch. As only 36.2% of posts in the training and validation sets had received at least one comment, we adopted a 2-stage fine-tuning strategy to incorporate comments as contextual information. In the first stage, we fine-tuned a RoBERTa-base classifier using only the comments. In the second stage, we continued fine-tuning the comment-pretrained classifier using one of the 3 textual inputs described earlier (eg, title+post). All experiments were run under 3 random seeds (123, 28, 51), and the results were averaged across runs and reported as mean (SD).

##### LLM Prompting

Given the success of LLMs and prompt engineering [38], we further explored whether they could outperform supervised approaches that relied on significantly smaller models. We experimented with 2 open-source instruction-tuned LLMs—Llama-3.1-8B-Instruct [35] and Qwen2.5-14B-Instruct [36]—in both zero-shot and few-shot inference settings without any parameter updates. Inference used greedy decoding with a maximum of 10 new tokens, and a fixed random seed (42) was set for reproducibility. Input was truncated to a maximum context length of 4096 tokens. In the zero-shot setting, the model received only the task instruction and the input text (title only, post only, or title+post). For the few-shot condition, we varied the number of in-context examples (1, 3, and 5) and found that a single example (1-shot) yielded the best overall performance; we therefore reported 1-shot results as our few-shot condition.

### Ethical Considerations

This research received a Not Human Research determination from the Institutional Review Board at the University of Kentucky. The study was determined not to require institutional review board review because it analyzed publicly available Reddit posts and comments obtained through Reddit’s API and did not involve intervention or interaction with individuals or the collection of private identifiable information. To safeguard user privacy, we avoided reporting usernames, post titles, URLs, or verbatim quotations that could enable reidentification.

## Results

### Language Differences Across Recovery Stages (RQ1)

Our results indicated statistically significant linguistic differences in posts at different recovery stages (Table 1).

**Table 1.** Linguistic feature comparisons across opioid use disorder recovery stages in posts from r/OpiatesRecovery (January 2014 to May 2022)^a^.

Linguistic feature	Stage 1^b^ vs stage 2^c^		Stage 1 vs stage 3^d^		Stage 1 vs stage 4^e^		Stage 2 vs stage 3		Stage 2 vs stage 4		Stage 3 vs stage 4	
	MD	*P* value	MD	*P* value	MD	*P* value	MD	*P* value	MD	*P* value	MD	*P* value
Tokens	80.78^f^	<.001	32.86^f^	<.001	9.76^f^	<.001	−47.92^f^	<.001	−71.02	.08	−23.10	.10
Joyful words	−0.003^f^	<.001	−0.011^f^	<.001	−0.013^f^	<.001	−0.008^f^	<.001	−0.01^f^	<.001	−0.002	.005
Positive words	−0.015^f^	<.001	−0.028^f^	<.001	−0.036^f^	<.001	−0.013^f^	<.001	−0.020^f^	<.001	−0.008^f^	.03
Negative words	0.011^f^	<.001	0.014^f^	<.001	0.027^f^	<.001	0.003	.22	0.015	<.001	0.013	.004
Trustful words	−0.002	.07	−0.008^f^	<.001	−0.010^f^	<.001	−0.007^f^	<.001	−0.008^f^	<.001	−0.001	.03
Dominant words	−0.008^f^	<.001	−0.16^f^	<.001	−0.16^f^	<.001	−0.15^f^	<.001	−0.16^f^	<.001	−0.007^f^	<.001
Painful words	0.001^f^	<.001	0.002^f^	<.001	0.003^f^	<.001	0.002^f^	<.001	0.003	.001	0.001	.09
Needful words	0.001	.003	0.003^f^	<.001	0.005^f^	<.001	0.002^f^	<.001	0.004	.001	0.002	.09
Fearful words	0.023^f^	<.001	0.015^f^	<.001	0.026	.001	−0.008^f^	<.001	0.003	.86	0.01	.14
Passive words	0.003^f^	<.001	0.004^f^	<.001	0.008^f^	<.001	0.001	.57	0.004	.04	0.003	.06

a Pairwise Mann-Whitney *U* tests report mean differences (MDs) and 2-tailed *P* values.

b Addiction.

c Initial recovery.

d Sustained recovery.

e Stable recovery.

f Results that remain significant after Bonferroni correction.

Regarding the number of tokens per post, users at the *addiction* stage (stage 1) used significantly more words than those at later stages of recovery. Specifically, compared with users in the *initial* recovery stage, the mean difference (MD) was 80.78 tokens (*P*<.001); compared with those in the *sustained* recovery stage, the MD was 32.86 tokens (*P*<.001); and compared with those in the *stable* recovery stage, the MD was 9.76 tokens (*P*<.001).

Regarding the ratio of sentiment and cognitive words, Reddit users in the earlier stages of recovery (*addiction* and *initial* recovery) showed lower proportions of trust-related, positive, and dominant words than those in later stages of recovery while exhibiting higher proportions of negative, pain-related, fear-related, and passive words. For example, the ratio of joyful words in posts by users at the addiction stage was significantly lower than that of users in the *sustained* recovery stage (MD −0.011; *P*<.001) and the *stable* recovery stage (MD −0.013, *P*<.001). These findings suggested that users in later recovery stages tended to express more positive and self-dominant emotions, experience less pain and passivity, and show a reduced reliance on others.

### Social Support for Posts at Different Recovery Stages (RQ2)

#### Correlation of Different Types of Social Support

**Figure 2.**
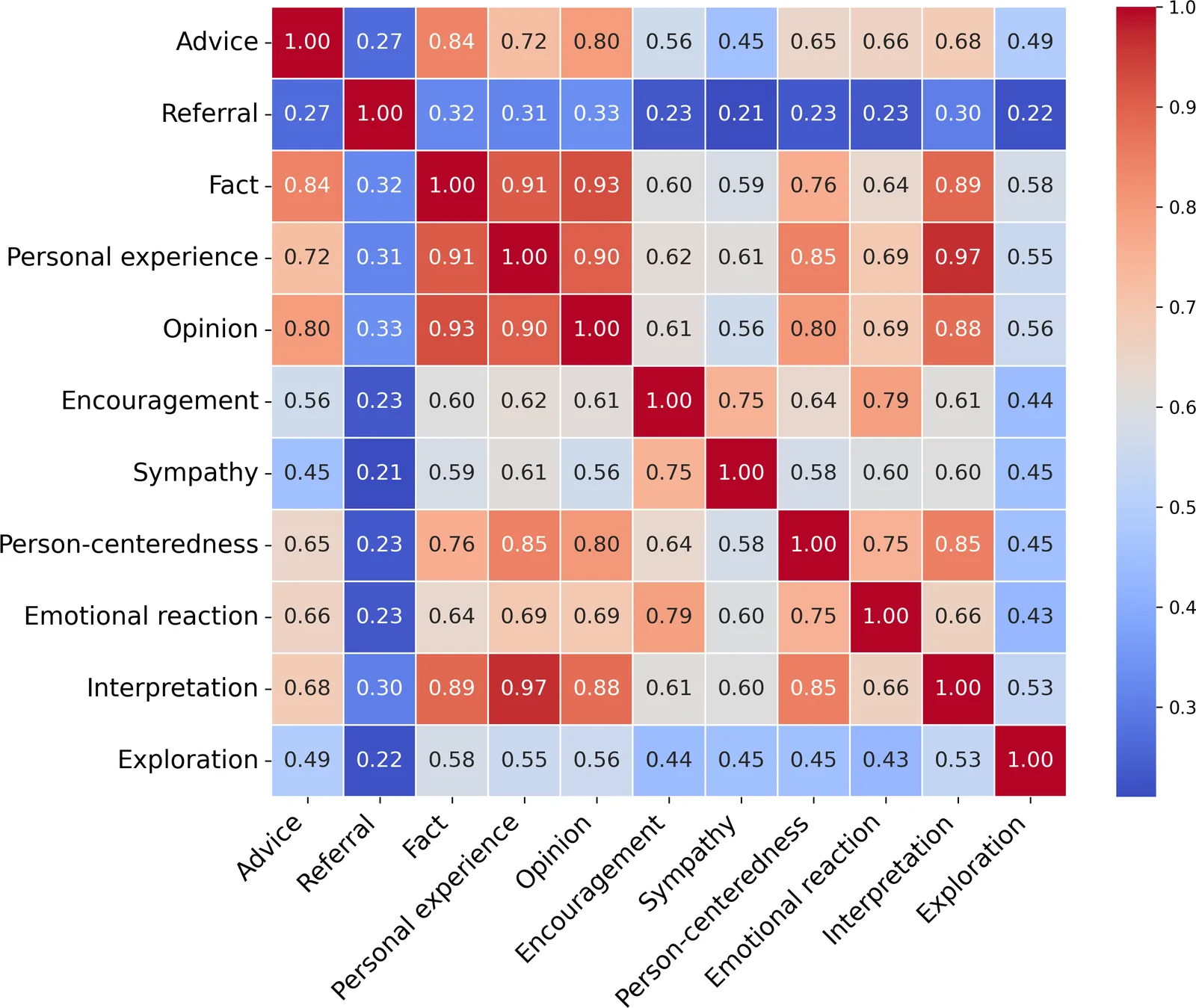
Heatmap of pairwise Pearson correlations among informational and emotional support subtypes in comments from r/OpiatesRecovery (January 2014 to May 2022). Correlations were aggregated at the post level. All correlations shown were statistically significant at *P*<.001.

To examine how different types of support were associated in responses to posts, we aggregated all comments associated with each post and computed pairwise correlations between support subtypes. All correlations shown were statistically significant at *P*<.001. As shown in Figure 2, certain support types were strongly correlated at the post level.

Among informational support subtypes, *fact*, *opinion*, and *personal experience* showed consistently high correlation (eg, *fact–opinion: r*=0.93), suggesting that Reddit users received a blend of factual advice, personal narratives, and evaluative input. Emotional support subtypes such as *encouragement*, *sympathy*, and *emotional reaction* were also positively correlated, especially with *person-centeredness* and *interpretation*, indicating the delivery of both empathy and cognitive reframing. Additionally, *person-centeredness* and *interpretation* bridged emotional and informational support, with high correlation with *personal experience*, *opinion*, and *fact*, while *referral* appeared more isolated, showing weak correlation with most types.

#### Differences in Informational Support

**Figure 3.**
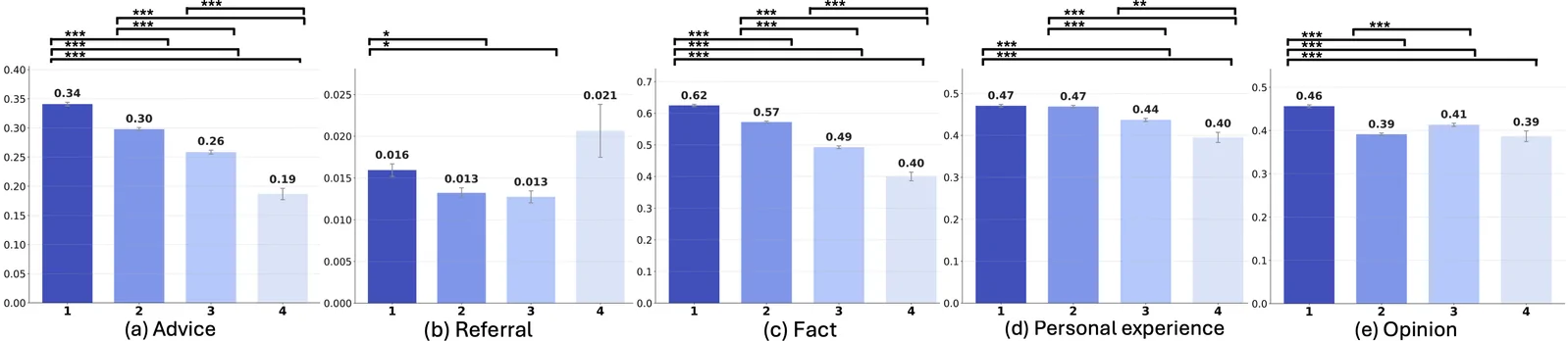
Informational support across opioid use disorder recovery stages in comments from r/OpiatesRecovery (January 2014 to May 2022). Results are based on one-way ANOVA with Games-Howell post hoc comparisons. Each subplot shows mean values across 4 recovery stages (1=addiction, 2=initial recovery, 3=sustained recovery, 4=stable recovery), with error bars indicating SEs. Advice: *F*_3,22845_=148.46***; *η*²=0.02; Referral: *F*_3,22845_=6.42***; *η*²<.01; Fact: *F*_3,22845_=259.70***; *η*²=0.03; Personal experience: *F*_3,22845_=29.89***; *η*²<.01; and Opinion: *F*_3,22845_=75.74***; *η*²=.01. *P<.05, **P<.01, ***P<.001.

Results from one-way ANOVA tests with Games-Howell post hoc comparisons suggested that posts featuring earlier recovery stages typically received more informational support than those featuring later recovery stages (Figure 3). This trend was particularly evident for *advice*, where *addiction*-stage posts prompted significantly more input than those made during the *initial* (MD 0.04; *P*<.001), *sustained* (MD 0.08; *P*<.001), and *stable recovery* stages (MD 0.15; *P*<.001). A similar pattern emerged for *referrals*, with *addiction*-stage posts receiving more *referrals* than those in the *initial* (MD 0.002; *P*=.02) and *sustained* stages (MD 0.003; *P*=.01). Similarly, posts from the *addiction* stage prompted significantly more facts than those from the *initial* (MD 0.05; *P*<.001), *sustained* (MD 0.13; *P*<.001), and *stable recovery* stages (MD 0.22; *P*<.001). It was also observed that posts in the *addiction* stage drew more *personal experiences* than those from the *sustained* (MD 0.03; *P*<.001) and *stable* stages (MD 0.08; *P*<.001). Finally, *addiction*-stage posts had received significantly more *opinions* than those from the later stages: *initial* (MD 0.06; *P*<.001), *sustained* (MD 0.04; *P*<.001), and *stable stages* (MD 0.07; *P*<.001).

#### Differences in Emotional Support

**Figure 4.**
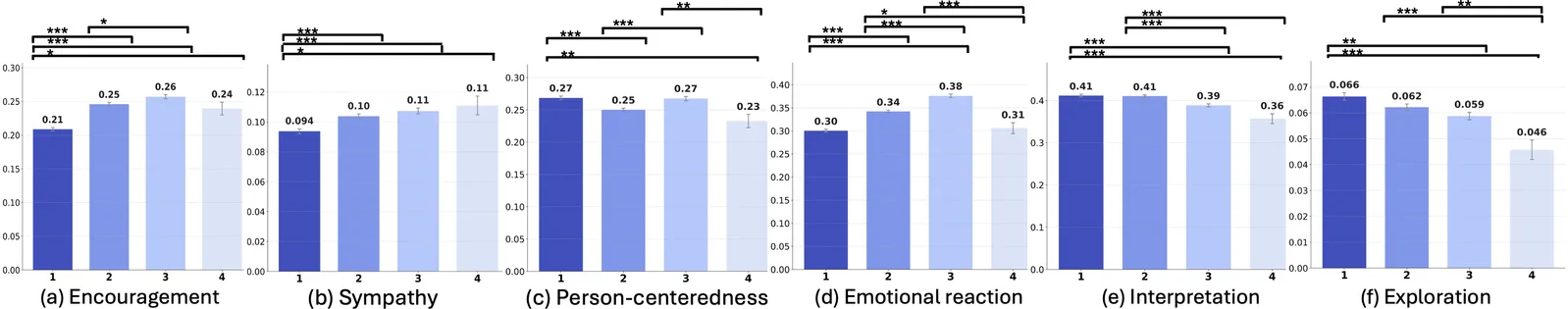
Emotional support across opioid use disorder recovery stages in comments from r/OpiatesRecovery (January 2014 to May 2022). Results are based on one-way ANOVA with Games-Howell post hoc comparisons. Each subplot shows mean values across 4 recovery stages (1=addiction, 2=initial recovery, 3=sustained recovery, and 4=stable recovery), with error bars indicating SEs. Encouragement: *F*_3,22845_=55.17***; *η*²=.01; Sympathy: *F*_3,22845_=11.37***; *η*²<.01; Person-centeredness: *F*_3,22845_=12.05***; *η*²<.01; Emotional reaction: *F*_3,22845_=84.92***; *η*²=.01; Interpretation: *F*_3,22845_=15.75***; *η*²<.01; and Exploration: *F*_3,22845_=8.67***; *η*²<.01. *P<.05, **P<.01, ***P<.001.

As shown in Figure 4, results from one-way ANOVA tests with Games-Howell post hoc comparisons suggested a nuanced pattern in the emotional support received across recovery stages. Unlike the patterns observed for informational support, posts in the *addiction* stage (stage 1) received less *encouragement*, *sympathy*, and *emotional reactions* compared to posts from other recovery stages. For *encouragement*, *addiction*-stage posts received significantly less than those from the *initial* (MD –0.04; *P*<.001), *sustained* (MD –0.05; *P*<.001), and *stable recovery* stages (MD –0.03, *P*=.01). Additionally, posts in the *initial recovery* stage received significantly less *encouragement* than those in the *sustained* stage (MD –0.01; *P*=.03; encouragement: stage 2 vs stage 3). A similar pattern was observed for *sympathy*. *Addiction*-stage posts received significantly less than posts in the later stages: *initial* (MD –0.01; *P*<.001), *sustained* (MD –0.01; *P*<.001), and *stable recovery* stages (MD –0.02; *P*=.04). As for *emotional reactions*, *addiction*-stage posts also received significantly fewer reactions than those from the *initial* (MD –0.04, *P*<.001) and *sustained* stages (MD –0.08; *P*<.001).

Some forms of emotional support followed a trend similar to informational support. For example, *addiction*-stage posts received more *interpretation*-related comments than those disclosing the *sustained* (MD 0.02; *P*<.001) and *stable* stages (MD 0.05; *P*<.001). Similarly, *addiction* stage posts received more *exploration*-related comments than posts disclosing *sustained* (MD 0.01; *P*=.001) and *stable recovery* stages (MD 0.02, *P*<.001). Both *initial* (MD 0.02, *P*<.001) and *sustained* (MD 0.01; *P*=.006) recovery stages also received significantly more *exploration* than the stable stage.

Among all types of emotional support, *person-centeredness* showed the most complex pattern. *Addiction*-stage posts received significantly more *person-centeredness* than those from the *initial* (MD 0.02; *P*<.001) and *stable recovery* stages (MD 0.04; *P*=.005). However, posts in the *initial* stage received significantly less *person-centeredness* than those in the *sustained* stage (MD –0.02; *P*<.001), and posts in the *sustained* recovery stage received more *person-centeredness* than those from the *stable* stage (MD 0.03; *P*=.007).

### Differences in Social Support Across Recovery-Stage Transitions (RQ3)

In this section, we showed the results from a Bonferroni post hoc comparison among the 3 transition groups: *Recovery Progression*, *No Change*, and *Recovery Regression*. A multivariate test using Pillai’s trace indicated significant differences across transition groups in linguistic features and social support (Pillai’s trace =0.01; *F*_30,14,940_=3.52; *P*<.001; partial *η*^2^=0.01). Notably, the observed effect sizes were generally small according to conventional effect size benchmarks [39], suggesting that the transition group explained only a modest proportion of variance in linguistic and social support features. In addition, because these results were observational and based on classifier-generated labels, the group differences should be interpreted as associations rather than evidence that linguistic features and social support contributed to subsequent recovery-stage changes.

In terms of linguistic features, post hoc comparisons showed that users in the *Recovery Progression* group wrote significantly longer posts than those in the *Recovery Regression* (MD 20.92, SE 7.67; *P*=.02) and *No Change* groups (MD 36.14, SE 6.27; *P*<.001). They also expressed fewer positive words (*Recovery Regression group:* MD −0.10, SE 0.02; *P*<.001; *No Change group:* MD −0.10, SE 0.02; *P*<.001) and more negative words (MD 0.08, SE 0.03; *P*=.002; *No Change group*, MD 0.05, SE 0.02; *P*=.02), suggesting that those progressing in recovery might have shared more emotionally vulnerable or difficult experiences.

**Figure 5.**
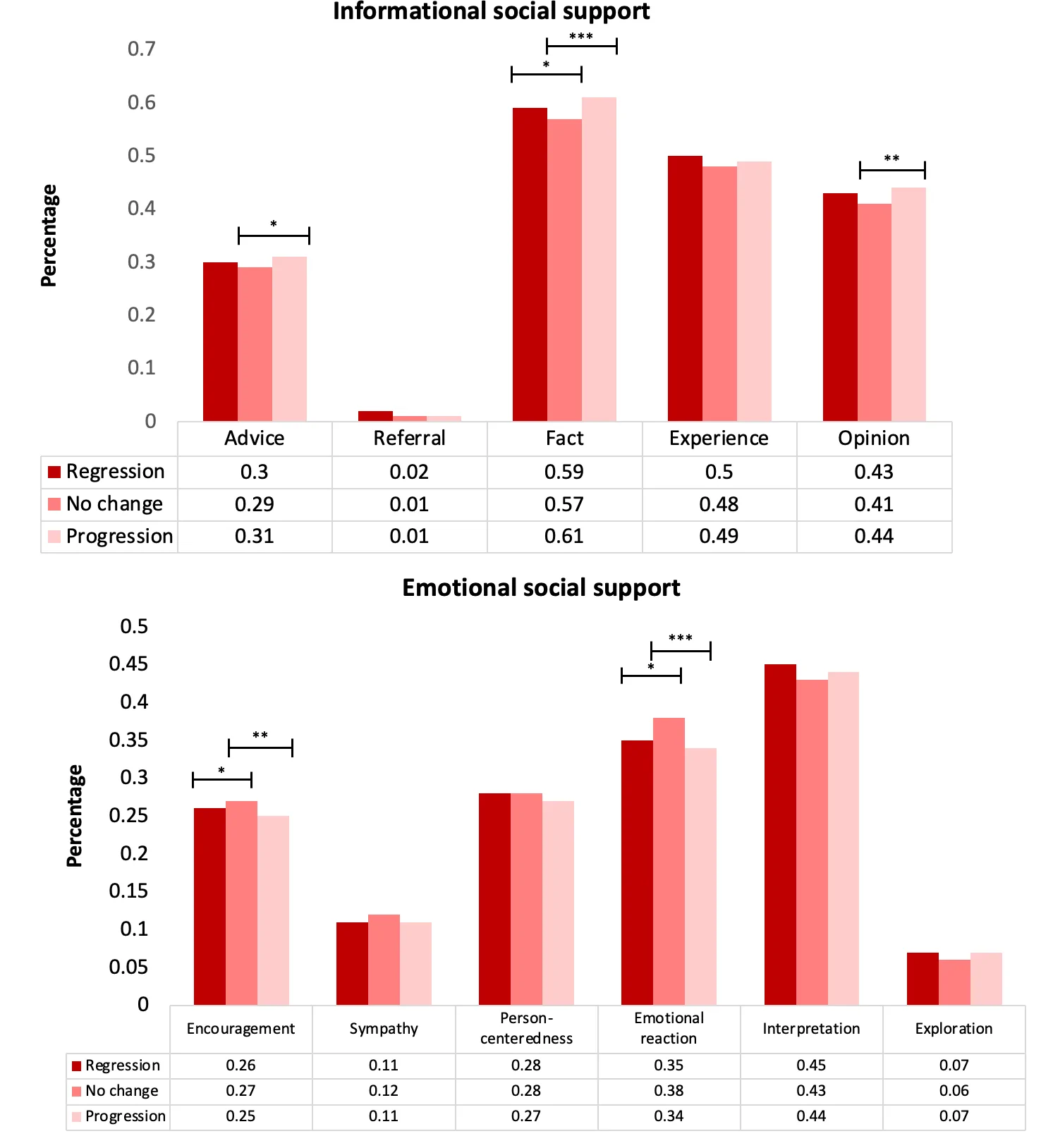
Informational and emotional support across recovery-stage transition groups in opioid use disorder posts from r/OpiatesRecovery (January 2014 to May 2022). The analysis included 8070 consecutive post pairs from 2936 users and compared recovery progression, no change, and recovery regression groups using multivariate analysis of covariance with posting interval as a covariate. *P<.05, **P<.01, ***P<.001.

Regarding informational support, the *Recovery Progression* group received significantly more *advice* (MD 0.02, SE 0.01; *P*=.03), *facts* (MD 0.04, SE 0.01; *P*<.001), and *opinions* (MD 0.02, SE 0.01; *P*=.006) than the *No Change* group (Figure 5). Additionally, the *Recovery Regression* group received more *factual* support (MD 0.02, SE 0.01; *P*=.05) than *No Change* users. These patterns suggested that transitions, both forward and backward, were associated with receiving greater informational support from the community.

In contrast, emotional support was more likely to be received by users in the *No Change* group. Specifically, they received more *encouragement* than both the *Recovery Regression* group (MD 0.02, SE 0.01; *P*=.04) and the *Recovery Progression* groups (MD 0.02, SE 0.01; *P*=.008). Similarly, they received more *emotional reactions* than those in the *Recovery Regression* (MD 0.02, SE 0.01; *P*=.03) and *Recovery Progression* groups (MD 0.03, SE 0.01; *P*<.001). These findings indicated that users who remained in the same recovery stage tended to receive more emotional support, such as encouragement and emotional reactions, than those undergoing transitions.

### Forecasting Recovery-Stage Transitions (RQ4)

Table 2 presents per-class and weighted average precision, recall, and *F*_1_-scores for all models and input configurations. Regarding supervised approaches, using only the post body as input already offered a solid improvement over the majority baseline (*F*_1_-score=0.55 vs 0.44). The post proved more informative than the title alone for forecasting transitions (*F*_1_-score=0.55 vs 0.49). The results improved further when using both the title and the post (*F*_1_-score=0.56 vs 0.55). Finally, the network that took both the title and the post and was pretrained with comments yielded the best results (*F*_1_-score=0.59). As these experiments were intended to establish an initial benchmark, we reported variability across random seeds but did not interpret small differences between RoBERTa configurations as statistically significant. Together, these results suggested that comments received on posts might provide useful contextual information when predicting individuals’ transitions to subsequent recovery stages, although this interpretation should be treated as preliminary. Across all configurations, *Recovery Progression* and *Recovery Regression* remained the most challenging classes, with models showing consistently low recall for these minority classes, while *No Change* was predicted more reliably across all settings.

Under the prompting settings evaluated here, both Llama-3.1-8B-Instruct and Qwen2.5-14B-Instruct, however, performed worse than the fine-tuned RoBERTa models on our task. Llama fell below the majority baseline across nearly all settings, with weighted *F*_1_-score ranging from 0.11 to 0.32. A consistent failure mode was overpredicting *Recovery Progression* with high recall but near-zero precision. Qwen performed somewhat better, with weighted *F*_1_-score ranging from 0.35 to 0.43, yet its best result (title few-shot, *F*_1_-score=0.43) still did not reach the majority baseline. Both models produced near-zero *F*_1_-score on *Recovery Regression* across almost all configurations. Our results were consistent with the findings in Yang et al [16], who focused on a related but different task, identifying the stages of OUD. Note that our task was more challenging: instead of identifying the current stages, we forecasted the future transitions, which required temporal reasoning beyond what off-the-shelf LLM prompting could support. These results should be interpreted as a comparison between fine-tuned supervised models and prompted open-source LLM baselines, rather than as evidence about the performance of LLMs after task-specific fine-tuning.

**Table 2.** Performance of language models forecasting future opioid use disorder recovery-stage transitions from current posts in r/OpiatesRecovery (January 2014 to May 2022; 8070 post pairs)^a^.

Configuration	Progression			No change			Regression			Average		
	Precision (SD)	Recall (SD)	*F*_1_-score (SD)	Precision (SD)	Recall (SD)	*F*_1_-score (SD)	Precision (SD)	Recall (SD)	*F*_1_-score (SD)	Precision (SD)	Recall (SD)	*F*_1_-score (SD)
Baseline	0.00	0.00	0.00	0.59	1.00	0.75	0.00	0.00	0.00	0.35	0.59	0.44
RoBERTa^b^ based												
title	0.40 (0.09)	0.19 (0.13)	0.23 (0.10)	0.63 (0.04)	0.85 (0.15)	0.71 (0.03)	0.12 (0.11)	0.08 (0.11)	0.09 (0.11)	0.49 (0.03)	0.56 (0.04)	0.49 (0.02)
+pretrain	0.41 (0.01)	0.37 (0.07)	0.39 (0.04)	0.65 (0.02)	0.75 (0.11)	0.69 (0.03)	0.14 (0.12)	0.12 (0.12)	0.13 (0.12)	0.51 (0.03)	0.56 (0.03)	0.53 (0.01)
post	0.47 (0.05)	0.36 (0.11)	0.41 (0.09)	0.64 (0.02)	0.81 (0.05)	0.71 (0.01)	0.26 (0.01)	0.10 (0.04)	0.14 (0.04)	0.54 (0.02)	0.58 (0.01)	0.55 (0.02)
+pretrain	0.51 (0.06)	0.27 (0.02)	0.35 (0.01)	0.63 (0.00)	0.86 (0.06)	0.73 (0.02)	0.35 (0.04)	0.13 (0.08)	0.18 (0.07)	0.56 (0.02)	0.60 (0.02)	0.55 (0.00)
title+post	0.52 (0.07)	0.32 (0.09)	0.39 (0.09)	0.66 (0.03)	0.80 (0.10)	0.72 (0.03)	0.32 (0.08)	0.22 (0.11)	0.24 (0.05)	0.57 (0.03)	0.59 (0.03)	0.56 (0.03)
+pretrain	0.54 (0.02)	0.40 (0.14)	0.45 (0.10)	0.70 (0.04)	0.73 (0.04)	0.71 (0.01)	0.30 (0.03)	0.36 (0.03)	0.33 (0.03)	0.60 (0.02)	0.59 (0.01)	0.59 (0.02)
Llama prompt												
title (zero shot)	0.24	0.79	0.37	0.58	0.09	0.16	0.05	0.03	0.04	0.41	0.25	0.19
title (few shot)	0.19	0.29	0.23	0.57	0.32	0.41	0.12	0.21	0.15	0.40	0.29	0.32
post (zero shot)	0.22	0.79	0.35	0.69	0.08	0.14	0.19	0.09	0.12	0.50	0.25	0.18
post (few shot)	0.22	0.71	0.33	0.83	0.04	0.08	0.11	0.12	0.11	0.57	0.21	0.15
title+post (zero shot)	0.23	0.85	0.36	0.67	0.02	0.03	0.00	0.00	0.00	0.45	0.21	0.11
title+post (few shot)	0.22	0.71	0.33	0.57	0.03	0.06	0.08	0.09	0.09	0.41	0.20	0.13
Qwen prompt												
title (zero shot)	0.16	0.19	0.17	0.59	0.62	0.60	0.05	0.03	0.04	0.40	0.42	0.41
title (few shot)	0.15	0.04	0.07	0.59	0.85	0.69	0.00	0.00	0.00	0.39	0.52	0.43
post (zero shot)	0.13	0.23	0.17	0.59	0.41	0.49	0.12	0.12	0.12	0.40	0.32	0.35
post (few shot)	0.11	0.17	0.14	0.60	0.57	0.58	0.06	0.03	0.04	0.39	0.39	0.39
title+post (zero shot)	0.18	0.35	0.24	0.66	0.39	0.49	0.09	0.09	0.09	0.45	0.34	0.37
title+post (few shot)	0.10	0.08	0.09	0.60	0.73	0.66	0.00	0.00	0.00	0.38	0.46	0.41

a For RoBERTa-based models, precision, recall, and *F*_1_-score are reported as mean (SD) across 3 random seeds. Baseline and prompted large language model results are reported from deterministic inference runs.

b RoBERTa: Robustly Optimized BERT Pretraining Approach.

## Discussion

### Principal Findings

This study investigated how nuanced forms of social support in online health communities correspond to different stages of recovery from OUD. We identified several key empirical findings. First, our linguistic analyses revealed that users’ language patterns (eg, emotional valence and dominance) varied significantly across recovery stages. Our findings indicated that Reddit users in the addiction stage expressed fewer joyful, positive, dominant, and trust-related words, but more negative, pain-related, and passive language. In contrast, users in more advanced recovery stages used more positive and assertive language, showing greater emotional positivity and agency. These results are consistent with prior linguistic studies on mental health recovery [8,40]. For example, De Choudhury and De [8] analyzed Reddit communities related to mental health and observed that users in earlier recovery stages often used inhibition-related words (eg, avoid, escape, or deny) to express their feelings of diminished agency and lack of control. While their study highlights the relationship between language and recovery, our work extends this literature by uncovering how language use evolves across distinct recovery stages over time. This observed longitudinal evolution is important because it allows for a more precise understanding of individuals’ recovery trajectories and can inform the design of tailored digital support systems.

In addition, we observed a stage-sensitive adaptation in the types of support offered by the community. Consistent with prior research [7,8,40,41], our findings demonstrate that online spaces such as *r/OpiatesRecovery* may provide settings in which stage-specific peer support can be observed and studied. Our statistical analysis further showed that Reddit users in the earlier stages of recovery received more informational support, such as *advice*, *referrals*, *facts*, *personal experiences*, and *opinions*, compared to those in later stages. In contrast, users in the sustained and stable recovery stages were more likely to receive *emotional reactions*, *sympathy*, and *encouragement*. These patterns suggest that the distribution of support types differs across posts classified into different recovery stages. This dynamic responsiveness has not been fully examined in prior substance recovery research and extends existing studies on online peer support [7,42], which often treated the recovery stage as a static or cross-sectional factor. In contrast, our study highlights how both language use and the nature of social support shift dynamically throughout the recovery process. These findings underscore the importance of recognizing recovery as a fluid, evolving journey and suggest that future peer-support systems should account for variation in support needs across stages.

In addition, although several multivariate and univariate effects reached statistical significance, the associated effect sizes were generally small according to conventional benchmarks for small effects, suggesting that transition groups explain a modest proportion of variance in linguistic and social support features. Nevertheless, the observed patterns were consistent across multiple support dimensions, indicating modest differences associated with recovery trajectories. Such effect sizes are common in large-scale observational psychological research, where behavioral outcomes are shaped by numerous interacting factors [43,44]. In the context of OUD recovery, predicting complex and internally experienced recovery transitions from naturally occurring social media discourse is inherently challenging, as language use captures only one multidetermined aspect of individuals’ lived experiences [18]. Thus, explaining even a small proportion of variance across a large corpus may reflect a subtle but reliable behavioral signal, offering meaningful population-level insight into recovery dynamics, even if these effects are insufficient for individual-level clinical prediction.

Notably, the pattern in which users in the addiction stage received less *sympathy* may reflect broader societal stigma toward people who use drugs. This stigma often stems from moralistic narratives that frame substance use as a personal failing or a sign of weak character, rather than as a complex health issue shaped by structural, psychological, and social factors [40]. As a result, individuals in the early stages of recovery may be perceived as less “deserving” of empathy or support than those who have already demonstrated progress or stability in their recovery journey. Our findings suggest that even within ostensibly supportive online spaces, implicit biases may shape how community members respond to individuals based on their perceived stage of recovery. Although our study does not establish a causal relationship, this observed pattern highlights the need to foster more inclusive and nonjudgmental support environments that recognize recovery as a nonlinear and deeply individualized process and affirm the dignity and humanity of people at all stages of that journey.

Finally, our longitudinal design revealed that recovery from OUD is not a linear or uniform process, but rather a dynamic and transitional one. User recovery trajectories on *r/OpiatesRecovery* reflected a range of patterns, including forward progress, setbacks, or relapses. These observed patterns extended periods of stasis, highlighting the inherently fluctuating nature of substance addiction recovery [45,46]. These observations align with prior findings from systematic reviews [47], which emphasize that recovery often unfolds in a cyclical rather than stepwise fashion. In this study, our results not only confirm the nonlinear structure of recovery but also map these shifts to evolving patterns of language and peer support. For instance, users in transition groups differed from those with no observed stage change in the informational and emotional support associated with their posts. These patterns suggest that observed recovery stage changes co-occur with differences in the types of responses users receive from the community, although the direction and mechanisms of these associations cannot be fully determined from the present data. By capturing these transitions over time, our findings underscore the need for flexible and adaptive support systems that recognize the instability and complexity of real-world recovery experiences. Rather than assuming a fixed stage or linear progression, future interventions, particularly in digital health and peer-based platforms, may need to accommodate setbacks, re-engagements, and plateaus as normative aspects of the recovery journey.

### Theoretical Contributions

Our study offers several key theoretical contributions to the literature on social support, substance use recovery, and online health communities. Importantly, although many observed differences were statistically significant, the associated effect sizes were generally small by conventional standards. This suggests that recovery stages represent only one of many factors shaping linguistic behavior and social support exchanges in online recovery communities. First, this study extends the social support theoretical model [10] by systematically integrating interdisciplinary perspectives from information science (eg, online information exchange behaviors), communication (eg, informational and emotional support types), and psychology (eg, emotional appraisal and sympathy). Most prior research has treated social support using limited typologies. In contrast, our work provides a comprehensive theoretical mapping of 11 distinct subtypes of social support across specific stages of recovery from OUD, suggesting that support may be understood as a stage-sensitive construct in the online recovery context. This multidimensional operationalization contributes to a theoretical refinement of the social support construct in digital health contexts.

Second, we propose a computational framework that reconceptualizes social support not as a static interaction but as a context-sensitive, evolving process that adapts to users’ shifting stages in recovery [42]. By analyzing longitudinal data from user-generated Reddit posts, our framework captures how observed support patterns and recovery stages vary over time. This approach addresses a key limitation in prior recovery and communication research, which often relies on cross-sectional or anecdotal evidence. The framework also offers theoretical generalizability, serving as a model for examining adaptive support in other behavioral health domains such as alcohol and tobacco cessation.

Third, our findings advance theories of online peer support and collective intelligence by showing how Reddit communities may function as informal peer-support environments in which response patterns vary by recovery stage. Specifically, we demonstrate that community members on *r/OpiatesRecovery* showed different support-type distributions across posts classified into different recovery stages. This finding expands our theoretical understanding of how peer-driven support is co-constructed and aligned with individual needs in digital health environments.

Finally, we contribute to addiction recovery theory by offering an empirically grounded, temporal model of recovery that foregrounds its nonlinear and transitional nature. We demonstrate that individuals’ trajectories reflect ongoing changes, including progressions, regressions, and stasis, in both their experiences and social support needs. This challenges traditional models that view recovery as a fixed outcome or single-stage event [7,16] and aligns with emergent frameworks that treat recovery as a fluid, iterative process [45,47]. Our work thereby contributes to a more granular, process-oriented theoretical lens for understanding how recovery unfolds over time in both personal and communal contexts.

### Practical Implications

This work has practical implications for future digital health research and recovery support design, but these findings should be interpreted as probabilistic population-level patterns rather than deterministic indicators of individual recovery outcomes.

First, our findings may inform the design and evaluation of recovery-focused digital tools. Our linguistic analysis reveals stage-specific language patterns—such as increased negative and painful expressions in early recovery and more positive, dominant language in advanced recovery. These insights may help researchers and designers explore adaptive features that respond to different recovery-related communication needs. For example, future mobile health apps could examine whether language-based indicators, after additional validation, can help guide tailored resources, messages, or support recommendations. Similarly, the machine learning classifiers developed in this study may serve as research tools for analyzing large-scale recovery discussions.

Second, our findings offer practical implications for health communication and peer-led campaign design. We observed that posts classified as early recovery stages received more informational support, whereas posts classified as later stages received more emotional support. This observed support matching pattern may inform the development of more nuanced prompts, campaigns, and peer moderation protocols that are sensitive to variation in recovery-related communication needs, enhancing both relevance and impact.

Third, this research offers implications for understanding communication needs across recovery trajectories. While clinical interventions are typically guided by standardized protocols, our findings emphasize the value of peer-driven, bottom-up insights grounded in shared lived experience. Future clinical communication research may examine whether these population-level patterns can inform how the timing, tone, and content of recovery-related communication vary across stages. For example, our results show that users whose posts were classified as recovery progression received more advice, factual information, and appraisals from peers than users whose stage remained unchanged. This pattern suggests that information-rich communication may be especially relevant to users undergoing observed recovery-stage transitions, although future work is needed to determine whether such communication contributes to recovery outcomes. Supported by prior research on online substance use interventions [42], these findings suggest that stage-sensitive communication may be a promising direction for designing recovery support messages that are more responsive to individuals’ changing needs.

### Limitations and Future Work

This study has several limitations. First, our analyses rely on self-reported data from a single online community. Although *r/OpiatesRecovery* is the largest subreddit dedicated to opioid recovery, the findings may disproportionately reflect the experiences of individuals who choose to post or respond within this community. In addition, Reddit users are not representative of the broader population of individuals with OUD, and platform-specific norms, demographics, anonymity, and self-selection may shape both what users disclose and the types of support they receive [48]. Second, self-reported information in online settings is not clinically verified and may not fully reflect users’ actual recovery status. Similarly, the social support examined in this study is confined to interactions occurring within this subreddit. Little is known about the additional support users may receive offline or in other online spaces. Future work incorporating longitudinal or mixed method approaches, such as survey-based measures or clinical validation, may provide a more comprehensive understanding of users’ recovery experiences and support networks. Third, our analyses rely on classifier-generated labels for both recovery stages and social support types. Although the classifiers achieved moderate to strong performance, they are not perfect, and classification errors may propagate into downstream linguistic, statistical, transition, and prediction analyses. This concern is particularly relevant for categories with lower classifier performance, such as initial recovery, sympathy, and encouragement. The annotation of recovery stages and social support types involves inherent subjectivity, as human judgments about recovery stage and support type are influenced by individual background knowledge, interpretive frames, and linguistic ambiguity [49]. Recovery-stage labels are especially challenging because users’ self-disclosures can be ambiguous, incomplete, or difficult to distinguish at the boundaries between adjacent stages. Disagreements in boundary cases may introduce noise into the labels used to train and evaluate our classifiers. Future work should consider iterative annotation protocols, adjudication procedures, and uncertainty quantification to better characterize and mitigate this source of variability. Accordingly, the findings should be interpreted as patterns based on computationally inferred labels rather than clinically verified recovery statuses or labels manually verified across the full corpus. Fourth, our linguistic and statistical analyses are correlational and should not be interpreted as evidence of causality. In particular, our study does not establish that social support from Reddit communities influences an individual’s recovery. OUD recovery is a complex process shaped by a variety of factors, including personal motivation for change, history of trauma, support from family, treatment access, local drug policies, and other social and clinical contexts. Future work could incorporate a broader range of social, interpersonal, and contextual factors to better understand how these factors are associated with recovery trajectories. Furthermore, the relatively small effect sizes observed in several analyses further suggest that recovery trajectories are influenced by many interacting psychological, interpersonal, and structural factors beyond online communication alone. Finally, because recovery transitions were inferred from consecutive observable posts rather than continuous behavioral tracking, intervals between posts varied across users. Although the posting interval was controlled for, the observed transitions likely reflect heterogeneous recovery trajectories unfolding over different timescales. Future research using finer-grained longitudinal designs may help clarify how temporal dynamics shape recovery progression and regression.

### Conclusions

This study contributes a theoretically grounded computational framework for analyzing recovery stages of OUD. We work with genuine user-generated data from an online health community on Reddit named *r/OpiatesRecovery*. Leveraging transformer-based deep learning models, the framework was used to identify (1) the current stage of recovery given by a post (eg, *initial recovery*), (2) whether comments received by a post provide informational or emotional support, and (3) if so, the specific type of informational or emotional support (eg, *advice*, *encouragement*). After categorizing the posts, we conducted linguistic analyses to better understand the language individuals use at various stages of recovery. By comparing the types of informational and emotional support across these stages, the study describes how support patterns vary across observed recovery stages. To further examine how the support individuals received at a given stage was associated with subsequent recovery-stage transitions, we analyzed users’ posting trajectories and examined the types of support across transition groups. Finally, we used both supervised learning techniques and prompt engineering approaches to predict future recovery transitions.

This study draws the following conclusions. First, the language people use at different recovery stages varies significantly. People use significantly fewer positive and dominant words in earlier recovery stages (eg, *addiction* and *initial recovery*) compared with those at later stages (eg, *stable recovery*), while using significantly more negative and painful words. Second, individuals in earlier recovery stages, such as during active addiction, received significantly more informational support (eg, *advice* and *facts*) compared to those in later stages. Surprisingly, the opposite trend emerged for emotional support—individuals received significantly less emotional support in the earlier stages than in the later ones. This result may reflect a modest tendency for community members to provide more encouragement to individuals who have already made some progress in recovery. Third, when examining individual recovery trajectories, we found statistically significant but generally small differences in social support across transition groups. Reddit users who progressed to a later stage received more informational support (eg, *advice*, *facts*, and *opinions*) than those whose recovery stage remained unchanged, whereas users who remained in the same stage received more *encouragement* and *emotional reactions* than those who transitioned to different stages, whether progressing or regressing. These effects were modest in magnitude but consistent across multiple support dimensions, suggesting stable yet limited associations between recovery trajectories and peer response patterns. Finally, predicting future recovery transitions remains a challenging task. Although adding comments to titles and posts improves model performance, the overall results remain modest. Thus, our findings highlight the complexity of modeling future recovery trajectories, while revealing subtle but consistent signals embedded in online recovery discourse.

## Supplementary material

10.2196/91054 Multimedia Appendix 1 Annotation disagreement, model configuration, and per-class performance.

## Abbreviations

BERT: Bidirectional Encoder Representations from Transformers
LLM: large language model
MANCOVA: multivariate analysis of covariance
MD: mean difference
OUD: opioid use disorder
RoBERTa: Robustly Optimized BERT Pretraining Approach
RQ: research question
SEANCE: Sentiment Analysis and Social Cognition Engine
