# Supplementary material for: Language, Social Support, and Recovery-Stage Transitions in Opioid Use Disorder on Reddit: Computational Analysis
Source: J Med Internet Res. 2026 Sep 17;28:e91054. doi: 10.2196/91054 (PMC13585015; doi:10.2196/91054)
Supplement: Supplementary file 1 [file jmir-v28-e91054-s001.docx]

**Multimedia Appendix 1. Annotation Disagreement, Model Configuration, and Per-Class Performance**

This appendix summarizes the annotation disagreement patterns, model configurations, and per-class or per-label performance for the classifiers used in the present study.

**Table S1. Distribution of initial annotator disagreements for recovery-stage coding.**

| **Disagreement type** | **Specific comparison** | **n** | **%** |
| --- | --- | --- | --- |
| Adjacent stages | Addiction vs initial recovery | 12 | 26.1 |
|  | Initial recovery vs sustained recovery | 8 | 17.4 |
|  | Sustained recovery vs stable recovery | 1 | 2.2 |
| Non-adjacent stages | Addiction vs sustained recovery | 5 | 10.9 |
|  | Initial recovery vs stable recovery | 1 | 2.2 |
| Specific stage vs “unknown” | Addiction vs unknown | 9 | 19.6 |
|  | Initial recovery vs unknown | 6 | 13.0 |
|  | Sustained recovery vs unknown | 4 | 8.7 |

**Table S2. Per-class and per-label classifier performance.**

| **Category** | **Class/label** | **Precision** | **Recall** | **F1** |
| --- | --- | --- | --- | --- |
| Opioid use and recovery stages | Addiction | 0.95 | 0.83 | 0.89 |
|  | Initial recovery | 0.64 | 0.75 | 0.69 |
|  | Sustained recovery | 0.85 | 0.93 | 0.89 |
|  | Stable recovery | 0.91 | 0.88 | 0.89 |
|  | Unknown stage | 0.64 | 0.85 | 0.73 |
| Informational support subtypes | Advice | 0.94 | 0.94 | 0.94 |
|  | Referral | 1.00 | 0.80 | 0.89 |
|  | Fact and situational appraisal | 0.66 | 0.95 | 0.78 |
|  | Personal experience | 0.85 | 0.96 | 0.90 |
|  | Opinion | 0.85 | 0.88 | 0.86 |
| Emotional support subtypes | Encouragement | 0.62 | 0.62 | 0.62 |
|  | Sympathy | 0.64 | 0.61 | 0.60 |
|  | Person-centeredness | 0.84 | 0.83 | 0.84 |
|  | Emotional reaction | 0.85 | 0.85 | 0.85 |
|  | Exploration | 0.96 | 0.95 | 0.96 |
|  | Interpretation | 0.86 | 0.86 | 0.86 |

**Table S3. Configuration and hyperparameters for label-generation classifiers.**

| **Task** | **Input** | **Model** | **Task type** | **Labels** | **Loss function** | **Optimizer** | | **Learning rate** | **Batch size** | **Max length** | **Epochs** | **Model selection** |
| --- | --- | --- | --- | --- | --- | --- | --- | --- | --- | --- | --- | --- |
| Recovery-stage classification | Posts | bert-base-uncased | Multi-class, single-label | 5 | CrossEntropyLoss | AdamW | | 2e-5 | 8 | 512 | 10 fixed epochs | Best validation F1 loaded at end of training |
| Informational support subtype classification | Comments | bert-base-uncased | Multi-label | 5 | BCEWithLogitsLoss | AdamW | | 2e-5 | 8 | 512 | 30 fixed epochs | Best validation F1 loaded at end of training |
| Emotional support subtype classification | Comments | roberta-base | Binary classifiers for each subtype | 6 binary labels | CrossEntropyLoss | | AdamW | 2e-5 | 8 | 512 | 10 fixed epochs with early stopping | Best validation F1 loaded at end of training; early stopping patience = 3 epochs |

**Table S4. Configuration and hyperparameters for recovery-transition prediction models.**

| **Model family** | **Model/configuration** | **Input configurations** | **Task type** | **Training or inference setup** | **Key hyperparameters** | **Evaluation** |
| --- | --- | --- | --- | --- | --- | --- |
| RoBERTa-based supervised model | roberta-base sequence classifier | Title only; post body only; title + post; each with or without comment-based pretraining | Multi-class, single-label: Recovery Progression, No Change, Recovery Regression | 85/15 train/validation split stratified by transition label; three random seeds (123, 28, 51); optional weighted cross-entropy tested but not used as main result | AdamW; learning rate 1e-5; weight decay 0.01; batch size 8; maximum 5 epochs; early stopping patience 3 based on validation loss | Precision, recall, and F1 reported in the main manuscript as mean (SD) across random seeds |
| Prompted open-source LLMs | Llama-3.1-8B-Instruct; Qwen2.5-14B-Instruct | Title only; post body only; title + post | Multi-class, single-label: Recovery Progression, No Change, Recovery Regression | Zero-shot and few-shot prompting without parameter updates; 1, 3, and 5 in-context examples tested, with 1-shot reported as the few-shot condition | Greedy decoding; do_sample=False; maximum new tokens=10; fixed random seed=42; maximum context length=4096 tokens | Precision, recall, and F1 reported in the main manuscript |
